# Inferior Olivary nucleus degeneration does not lessen tremor in essential tremor

**DOI:** 10.1186/s40673-018-0080-3

**Published:** 2018-01-15

**Authors:** Elan D. Louis, Daniel Trujillo Diaz, Sheng-Han Kuo, Shi-Rui Gan, Etty P. Cortes, Jean Paul G. Vonsattel, Phyllis L. Faust

**Affiliations:** 1 Department of Neurology, Yale School of Medicine, Yale University, 15 York Street, PO Box 208018, New Haven, CT 06520-8018 USA; 2 Department of Chronic Disease Epidemiology, Yale School of Public Health, Yale University, New Haven, CT USA; 3 Center for Neuroepidemiology and Clinical Neurological Research, Yale School of Medicine, Yale University, New Haven, CT USA; 4 Department of Neurology, College of Physicians and Surgeons, Columbia University, New York, NY USA; 5 Department of Neurology and Institute of Neurology, First Affiliated Hospital, Fujian Medical University, Fuzhou, China; 6 Department of Pathology and Cell Biology, Columbia University Medical Center and the New York Presbyterian Hospital, New York, NY USA; 7 Taub Institute for Research on Alzheimer’s Disease and the Aging Brain, Columbia University, New York, NY USA

**Keywords:** Essential tremor, Cerebellum, Inferior olivary nucleus, Neurodegenerative, Purkinje cell, Pathology

## Abstract

**Background:**

In traditional models of essential tremor, the inferior olivary nucleus was posited to play a central role as the pacemaker for the tremor. However, recent data call this disease model into question.

**Case presentation:**

Our patient had progressive, long-standing, familial essential tremor. Upper limb tremor began at age 10 and worsened over time. It continued to worsen during the nine-year period he was enrolled in our brain donation program (age 85 – 94 years), during which time the tremor moved from the moderate to severe range on examination. On postmortem examination at age 94, there were degenerative changes in the cerebellar cortex, as have been described in the essential tremor literature. Additionally, there was marked degeneration of the inferior olivary nucleus, which was presumed to be of more recent onset. Such degeneration has not been previously described in essential tremor postmortems. Despite the presence of this degeneration, the patient’s tremor not only persisted but it continued to worsen during the final decade of his life.

**Conclusions:**

Although the pathophysiology of essential tremor is not completely understood, evidence such as this suggests that the inferior olivary nucleus does not play a critical role in the generation of tremor in these patients.

## Background

Essential tremor (ET) is one of the most common movement disorders and it is the most common tremor disorder of humans [1, 2]. Despite its high prevalence, disease mechanisms are not completely understood [3, 4]. In recent years, clinical [5–9] and neuroimaging [3, 10–12] studies suggest that the cerebellum plays an important role in the generation of tremor in ET. Furthermore, a constellation of pathological changes is present in the ET cerebellum, mainly in the cerebellar cortex and involving the Purkinje cell and its neighboring neuronal populations, and distinguishes ET from control brains. These findings further support the central role that the cerebellum plays in ET pathogenesis [13–17].

It is clear that the cerebellum is of mechanistic importance in ET. The disease is likely the result of an abnormal motor loop or motor network that originates in the cerebellar cortex and then in a downstream fashion involves the deep cerebellar nuclei, the motor nuclei of the thalamus and motor cerebral cortex (i.e., an abnormal cerebellar-thalamic-cortical loop). In older models of ET, the inferior olivary nucleus (ION) was also included, and it was this structure that was posited to drive the tremor. However, this older model has fallen out of favor [18, 19] for a number of reasons, including the normal appearance of the ION on most neuroimaging studies [3, 20–22] as well as on postmortem comparisons of ET and control brains [23].

Recently, Elkouzi and colleagues [24] reported a patient with longstanding ET who developed hypertrophic olivary degeneration later in life, characterized clinically by ataxia and palatal tremor and by olivary pseudo-hypertrophy on magnetic resonance imaging. His ET tremor did not lessen despite the onset of this second syndrome of olivary degeneration and, based on this observation, the authors concluded that the ION was not the source of tremor in their patient [24]. We now report a case with longstanding, severe familial ET. Postmortem examination revealed marked olivary degeneration in addition to cerebellar degenerative changes. Despite this olivary degeneration, the patient’s tremor had continued to worsen, rather than disappearing, during the last decade of life.

## Case presentation

### Clinical history

At age 85 years, a right-handed Caucasian man with a Master’s degree enrolled in the Essential Tremor Centralized Brain Repository, a joint effort between investigators at Yale and Columbia Universities [25, 26]. Upon enrollment, the patient signed a written informed consent form, which was approved by both the Yale and Columbia University ethics committees. He reported that tremor had begun at 10 years of age. There was a family history of tremor in two first-degree relatives - both his mother and brother had had tremor. He reported that he had never been exposed to medications with known cerebellar toxicity (e.g., diphenylhydantoin, cytosine arabinoside) nor had he been a heavy drinker of ethanol (as defined previously) [14, 27] at any point in time. He had been diagnosed with ET by a neurologist “many years” prior to enrollment. He had taken medications for the tremor at some point long before enrollment but had discontinued them and could not recall their names. His past medical history was otherwise notable for arthritis, diverticula, high blood pressure, an enlarged prostate and need for a hearing aid. Recently, he had required a cane to walk.

Upon enrollment, he reported that the tremor affected his arms rather than his head, jaw or voice and that it impaired his ability to perform many of his activities of daily living (e.g., carrying a cup of water or coffee, drinking from a glass, using a spoon to eat soup, and cutting, trimming and filing his finger nails). He declined a detailed videotape neurological examination at that time.

Three years after enrollment, at age 88, a detailed videotaped neurological examination was first performed and tremor was rated by one of the authors (E.D.L.), a senior movement disorders neurologist, from 0 to 3, as described [14, 28]. There was no tremor at rest. Right arm/left arm tremor ratings were as follows, respectively: 2/1.5 (arm extension), 3/1.5 (pouring water between two cups), 2/2 (finger-nose-finger maneuver), and 3/2 (drawing Archimedes spirals). During finger-nose-finger maneuver intention tremor was not visualized. Tremor was not visualized in the neck, voice, or jaw. Palatal tremor was not evident. There was no dysarthria or dysmetria of hand movements. There was mild dysmetria on the heel-shin test on both sides (Scale for the Assessment and Rating of Ataxia score = 1) [29]. There was no hypomimia, hypophonia, or reduction in arm swing, and Unified Parkinson’s disease Rating Scale [30] scores on finger taps, opening and closing fists, and wrist pronation-supination were in the 0 – 1 range in both arms. There was no dystonia. Motor timing disturbances were not evaluated. The gait was measured, cautious and slow but not ataxic; it was assisted with a cane. Because of the cane, the patient was reluctant to attempt tandem gait. Based on this history and examination, a Washington Heights Inwood Genetic Study of ET diagnosis of definite ET [31] was assigned.

**Fig. 1:**
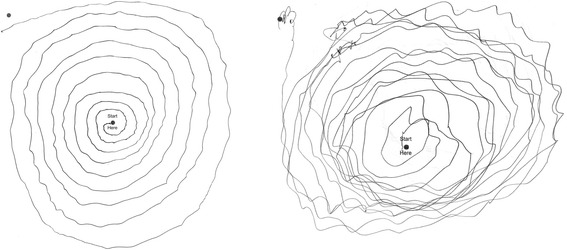
The patient’s Archimedes spirals (right arm), worsened over time - tremor rating = 2 (moderate) at age 85 (left panel) and tremor rating = 3 (severe) at age 93, which was 8.5 months prior to death (right panel)

During follow-up, his Archimedes spirals continued to show considerable tremor, which worsened over time - right/left ratings of 2/2 (age 85) evolved to 3/3 (age 93 [8.5 months prior to death]) (Fig. 1). Detailed discussions with his family indicated that the severe arm tremor persisted to the time of death at age 94.

A Folstein mini-mental state [32] score was 30/30 at age 88. A telephone interview for cognitive status [33] score was 32 /41 (age 89) and 29/41 (age 90). He died at age 94.

### Postmortem methods

As described [16, 34], the brain had a complete neuropathological assessment at the Essential Tremor Centralized Brain Repository in the New York Brain Bank and had standardized measurements of brain weight (grams) and postmortem interval (PMI, hours between death and placement of brain in a cold room or upon ice). Blocks were taken from standardized brain regions and embedded in paraffin; 7 μm-thick sections were stained with Luxol fast blue counterstained with hematoxylin-eosin (LH&E). Additional sections from selected blocks were stained with modified Bielschowsky silver stain, and others, with antibodies directed against α-synuclein (1:40, Leica, Buffalo Grove, IL, USA) (including cerebral cortex, cingulate gyrus, hippocampal formation, globus pallidum, putamen, amygdala, subthalamic nucleus, midbrain with substantia nigra, pons with the locus ceruleus, and medulla with the dorsal vagal nucleus), β-amyloid (1:400, Biocare Medical, Concord, CA, USA), and hyperphosphorylated tau (AT8) (1:200, Thermoscientific, Rockford, IL, USA), performed in an automated immunostainer (Ventana, Benchmark Ultra, Tuscon, AZ). Braak and Braak AD staging for neurofibrillary tangles [35, 36] and the Consortium to Establish a Registry for AD (CERAD) ratings for neuritic plaques [37] were assigned.

A standard 3 × 20 × 25 mm parasagittal, formalin-fixed, tissue block was harvested from the neocerebellum; this block included cortex, white matter and dentate nucleus [25, 26]. A senior neuropathologist (P.L.F.), blinded to clinical information, counted torpedoes in one entire LH&E section and another entire Bielschowsky-stained section [16] from that block. The neuropathologist counted heterotopic Purkinje cells (i.e., a Purkinje cell whose cell body was completely surrounded by the molecular layer and that did not contact the granule layer) in the entire LH&E stained section.

Purkinje cells in 15 randomly-selected 100× LH&E stained fields of the standard cerebellar section were counted. The average of these 15 counts was reported as the mean number of Purkinje cells per 100× field.

A semi-quantitative rating of the appearance of the basket cell plexus surrounding Purkinje cell bodies throughout the Bielschowsky preparation, described above, was applied using the following scale: 0 (few or no discernible processes); 1 (sparse number of processes); 2 (moderate number of processes); and 3 (dense tangle of processes), as described [17].

The density of neurons in the principal ION was quantified, counting only those neurons with an identifiable nucleolus in order to provide a point-like cell identifier. The total number of neurons with nucleoli was normalized to a traced linear length of the ION nucleus (in microns), measured with Neurolucida software (MicroBrightField Bioscience, Williston, VT). This was used to create an index of ION linear density (neurons/length in microns × 1000).

Calbindin_D28k_ immunohistochemistry was performed in free-floating 100 μm thick, formalin-fixed vibratome sections of cerebellar cortex to visualize Purkinje cell axonal morphology as described previously [14]. Purkinje cell axonal morphology in 10 randomly-selected 100× images was quantified: thickened Purkinje cell axonal profiles (an axon with at least double the width of other apparently normal axons), Purkinje cell axonal branching (any Purkinje cell axon with at least one branch point; multiple bifurcations on the same axon were not separately counted), and Purkinje cell axonal recurrent collaterals (an axon with at least a 90° turn back towards the Purkinje cell layer from its initial trajectory) [14]. Arciform axons were Purkinje cell axons that were more gradually curving back towards the Purkinje cell layer with a changing trajectory that was <90° [14]. Terminal axonal sprouting was the presence of a frayed terminal axonal region, often with a kinky appearance [14]. The raw counts of Purkinje cell axonal features were normalized to the total length of the Purkinje cell layer length (in microns) [14, 25, 26].

Seven μm thick paraffin-embedded sections of cerebellum and medulla were incubated with polyclonal rabbit anti-calbindin_D28k_ (1:1000, Swant Inc., Marly, Switzerland) or mouse monoclonal glutamatic acid decarboxylase (GAD) (1:100, MBL International, Woburn, MA) at 4 °C overnight after antigen retrieval in Trilogy (Cell Marque) in a vegetable steamer for 40 min, 100 °C. The sections were subsequently incubated with biotinylated anti-rabbit or anti-mouse IgG (1:200, Vector Labs, Burlingame, CA), and the signals were amplified by avidin/biotinylated complex with diaminobenzidine staining (Vector Labs).

### Postmortem findings

The brain, which weighed 1028.9 g, was placed on ice 3 h after death. External examination (J.P.G.V.) revealed mild to moderate cerebral cortical atrophy, mild amygdala atrophy, and moderate volume loss of the hippocampal formation. There was moderate to severe atrophy in the cerebellar vermis bilaterally, involving the entire superior vermis as well as the nodulus but with preservation of the remaining inferior vermis. Mild to moderate cerebellar cortical folial atrophy was evident bilaterally in the neocerebellum, variably involving both anterior and posterior lobes. The cerebellar dentate nucleus was grossly unremarkable. The remainder of the brain was grossly unremarkable.

On microscopic examination (J.P.G.V.), there were Alzheimer’s disease neuropathological changes, with parenchymal amyloid burden of deposits = A2, Braak & Braak stage for neurofibrillary tangles of Alzheimer = IV/VI (i.e., B2) and CERAD score for neuritic plaques = C1. Otherwise, the striatum, thalamus, and red nucleus were normal. On alpha synuclein immunostain, one 100× microscopic field of the basal region of the amygdala included five Lewy bodies but otherwise Lewy body containing neurons were rare in the amygdala. No Lewy body containing neurons were found elsewhere; that is, they were not found in the substantia innominata, substantia nigra pars compacta, locus ceruleus, or dorsal vagal nucleus on alpha synuclein immunostain.

**Fig. 2:**
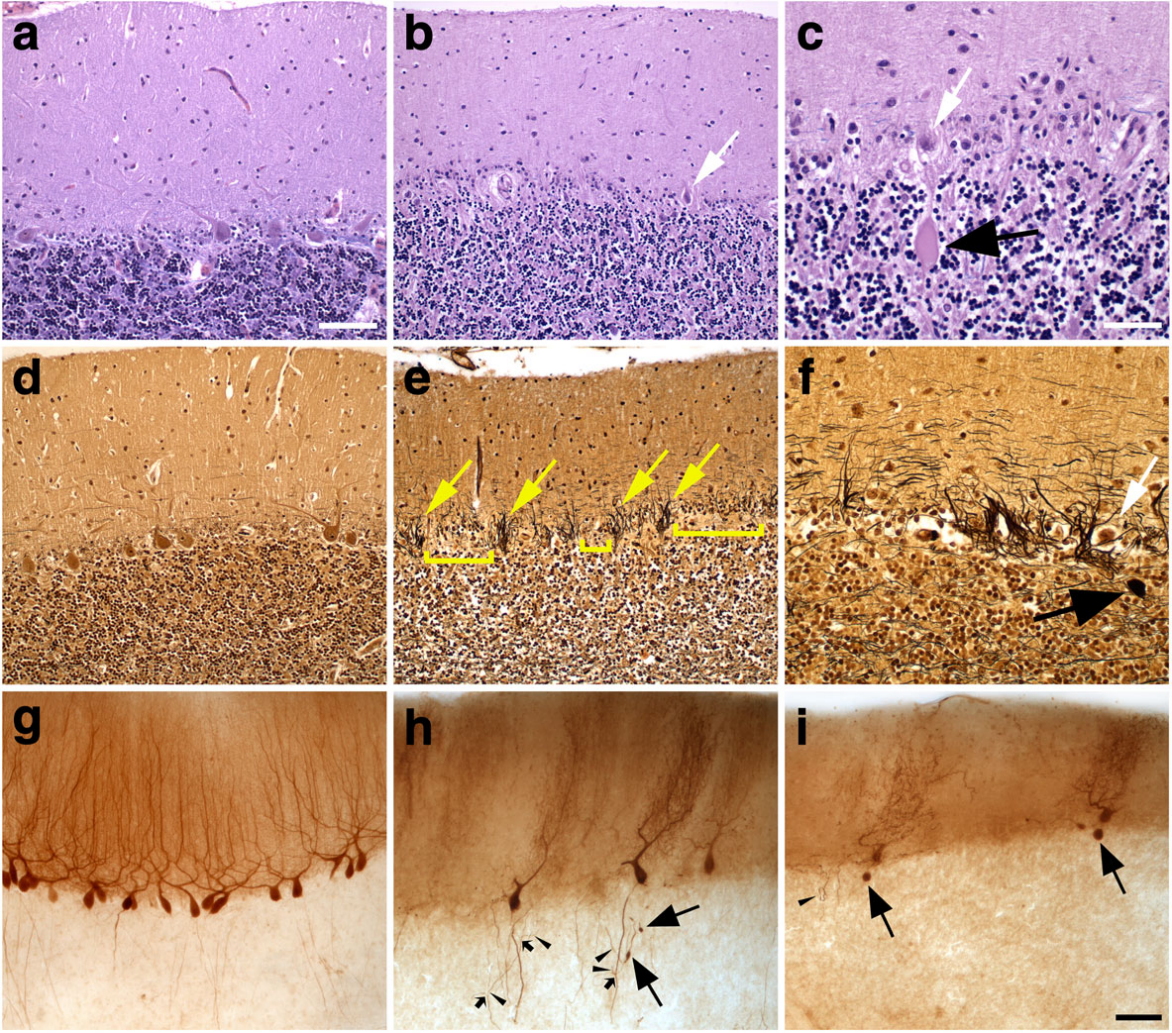
Degenerative changes in the patient’s cerebellar cortex (**b**, **c**, **e**, **f**, **h**, **i**) compared with a control (**a**, **d**, **g**) with LH&E stain (**a**-**c**), Bielschowsky stain (**d**-**f**) and calbindinD_28k_ immunostain (**g**-**i**). The molecular layer is thinner (**b**) and there is marked Purkinje cell loss with scattered, often atrophic Purkinje cells (**b**, **c**, white arrows) and Bergmann gliosis (**b**, **c**). Basket cell processes are coarse (**e**, yellow arrows), often around extant or atrophic (**f**, white arrow) Purkinje cells, and basket cell processes may be largely absent (**e**, yellow bars). Compared with control Purkinje cells (**g**), the patient’s Purkinje cell dendritic arbors are often stunted (**h**, **i**) and axonal changes including torpedoes (**c**, **f**, **h**, **i**, large black arrows), axonal branching (H, small black arrows) and recurrent collaterals (**h**, **i**, carets) are increased. Scale bars: 100 μm, A, B, D, E; 50 μm, C, F; 100 μm, G-H

The cerebellar cortex was characterized by a range of degenerative changes (Fig. 2), as have been described in patients with ET. Purkinje cell loss was marked, and accompanied by marked or focally severe Bergmann gliosis (Fig. 2a-c; Table 1). The molecular layer was thinner than normal. The loss of granular neurons was patchy, with a tendency to more severely affect the tips of folia than their depths. There was an increase in torpedoes on both LH&E and Bielschowsky-stained sections and marked hypertrophy of basket cell axonal processes along with areas where basket cell processes were absent (Fig. 2c-f; Table 1). An abundance of several other Purkinje cell axonal changes was identified by calbindinD_28k_ immunohistochemistry (esp. axonal branching and axonal recurrent collaterals; Fig. 2g-i, Table 1), as well as an increase in Purkinje cell dendritic swellings on LH&E and Bielschowsky-stained sections (Table 1).

**Table 1:** Postmortem cerebellar changes in our patient

Measure	Data from current case^a^	Contextual comments (i.e., published comparative data)
Cerebellar Cortex		
Torpedoes (LH&E)	17	In a prior publication [16], the mean value in ET cases = 10.5 ± 8.0 and in controls = 1.7 ± 1.4. None of the 21 controls had a value >5.
Torpedoes (Bielschowsky)	19	In a prior publication [16], the mean value in ET cases = 16.5 ± 14.2 and in controls = 3.3 ± 7.3.
Axonal changes (calbindin_D28k_)		In a prior publication [14]:
Thickened axonal profiles	1.2 (0.8)	0.6 ± 0.7 (cases) vs. 0.4 ± 0.6 (controls)
Axonal branching	10.8 (7.5)	0.3 ± 0.4 (cases) vs. 0.1 ± 0.2 (controls)
Axonal recurrent collaterals	16.8 (11.7)	1.0 ± 0.8 (cases) vs. 0.4 ± 0.5 (controls)
Arciform axons	0.10 (0.07)	0.08 ± 0.11 (cases) vs. 0.04 ± 0.07 (controls)
Terminal axonal sprouting	0.13 (0.09)	0.19 ± 0.22 (cases) vs. 0.07 ± 0.14 (controls)
Dendritic swellings (LH&E)	3	In a prior publication [44], the mean value in 20 ET cases = 1.50 ± 1.79 and in 19 controls = 0.05 ± 0.23. None of the 19 controls had >1 dendritic swelling.
Dendritic swellings (Bielschowsky)	3.5	In a prior publication [44], the mean value in 20 ET cases = 2.70 ± 3.10 and in 19 controls = 0.37 ± 0.50. None of the 19 controls had >1 dendritic swelling.
Purkinje cell count (LH&E, 15 100× fields)	0.8	In a prior publication [16], the mean value in ET cases = 7.2 ± 2.6 and in controls = 9.6 ± 3.4. None of the 33 ET cases or 21 controls had values as low as 0.8.
Heterotopic Purkinje cells (LH&E)	1.5	In a prior publication [46], the median number in 35 ET cases = 3 and in 32 controls = 1.
Basket rating (Bielschowsky)	3	In a prior publication [17], 8/37 (21.6%) ET cases and 3/69 (4.3%) controls had this value.
ION		
ION neuronal linear density (LH&E), neurons/μm × 1000	2.3	In a prior publication [23], the mean value in 14 ET cases = 9.4 ± 3.2 and in 15 controls = 8.8 ± 3.1.
Dentate Nucleus		
Dentate dorsal neuronal density, LH&E), neurons/μm^2^ × 10^−5^	1.95	In a prior publication [55], the mean value in 25 ET cases = 1.5 ± 0.5 and in 25 controls = 1.5 ± 0.4.
Dentate ventral neuronal density (LH&E), neurons/μm^2^ × 10^−5^	1.7	In a prior publication [55], the mean value in 25 ET cases = 1.5 ± 0.5 and in 25 controls = 1.4 ± 0.5.
Dentate total neuronal density, (LH&E), neurons/μm^2^ × 10^−5^	1.8	In a prior publication [55], the mean value in 25 ET cases = 1.5 ± 0.4 and in 25 controls = 1.4 ± 0.5.

*ET* Essential tremor, *ION* Inferior olivary nucleus, *LH&E* Luxol fast blue counterstained with hematoxylin-eosin

^**a**^Values are mean (median)

**Fig. 3:**
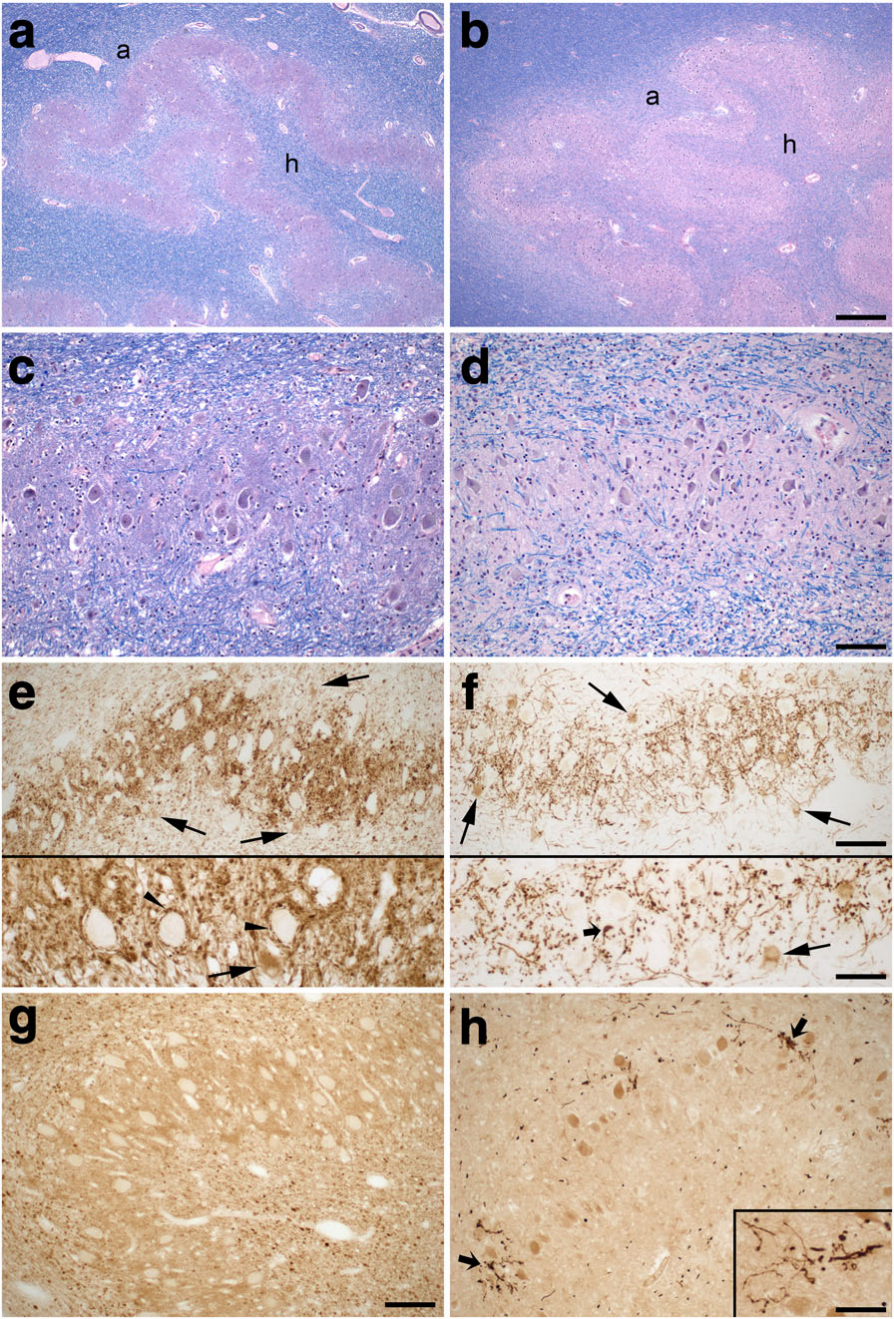
Degenerative changes in the patient’s dentate nucleus (**b**, **d**, **f**, **h**) compared with a control (**a**, **c**, **e**, **g**) with LH&E stain (**a**-**d**), GAD immunostain (**e**-**f**) and calbindinD_28k_ immunostain (**g**-**h**). **a**, **b** Myelin pallor in the patient’s amiculum (a) and hilum (h) of the dentate nucleus. **c**, **d** Dentate neuronal density is normal, although the soma of these neurons was often atrophic (**d**). Marked reduction of afferent synaptic density from Purkinje cells in dentate demonstrated by GAD (**e**, **f**) and calbinidinD_28k_ immunostain (**g**, **h**). GAD + −synaptic puncta around large GAD-negative dentate neurons (**e**, carets in bottom panel) are reduced (**f**), and some residual synaptic puncta are enlarged (F, small black arrow in bottom panel). GAD + −dentate neurons (**e**, **f**, black arrows) are readily identified. Degeneration and/or sprouting of residual Purkinje cell axons is evident (**h**, small black arrows and inset). There is artifactual staining of atrophic dentate neurons (**h**). Scale bars: 500 μm, A, B; 100 μm, C, D, and E, F (top panel), G, H. 50 μm, E, F (bottom panel), H, inset

The myelin stain of the amiculum and hilus of the dentate nucleus was weaker than normal; in contrast, the myelin stain of the adjacent album cerebelli was relatively spared (Fig, 3a and b). The neuronal density of the dentate nucleus was normal (Fig. 3c and d; Table 1); however, the soma of these neurons was atrophic. The normal dense synaptic staining of dentate by GAD antibodies that labels predominantly GABA-ergic Purkinje cell afferents, including distinct puncta around large GAD-negative neurons (Fig. 3e; carets in lower panel), was markedly reduced in the patient’s dentate (Fig. 3f), consistent with the marked loss of Purkinje cells; occasional perineuronal puncta were enlarged in size (Fig. 3f, lower panel, small arrowhead), suggesting synaptic degeneration. GAD+ GABA-ergic neurons in the dentate, which project to the inferior olive [38, 39], were readily detected in the patient’s dentate (Fig. 3e and f, arrows). As some of the GAD+ axons still seen in the patient’s dentate may be from processes of neurons intrinsic to the dentate, immunostain to calbindin_D28k_ was also performed, further demonstrating marked synaptic loss from Purkinje cells and only scattered degenerate and/or sprouting axonal profiles in the patient’s dentate (Fig. 3g and h).

**Fig. 4:**
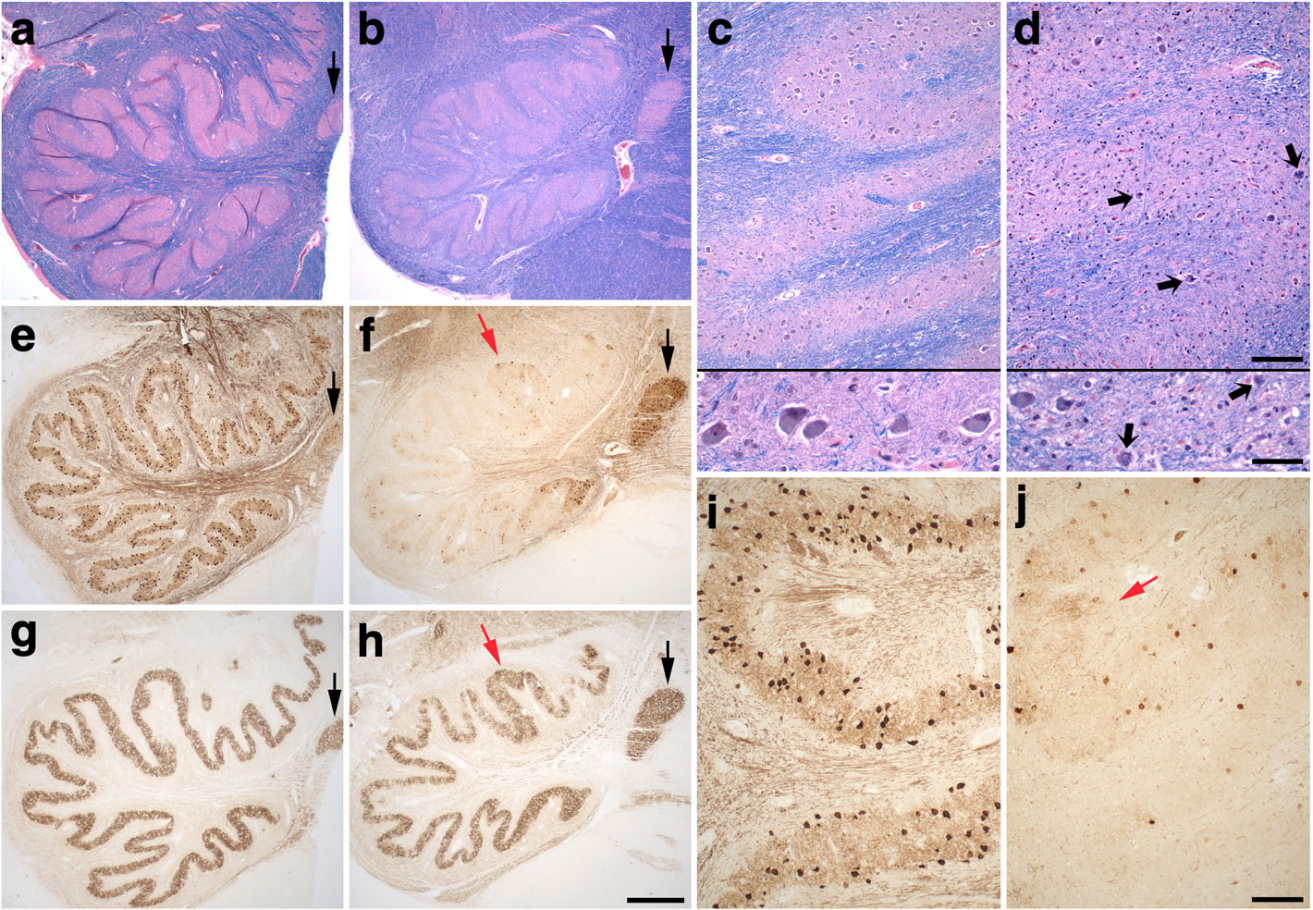
Degenerative changes in the patient’s ION (**b**, **d**, **f**, **h**, **j**) compared with a control (**a**, **c**, **e**, **g**, **i**) with LH&E stain (**a**-**d**), calbindinD_28k_ immunostain (**e**, **f**, **i**, **j**) and GAD immunostain (**g**, **h**). Marked patchy neuronal loss in the patient’s principal olive (**b**, **d**, **f**, **j**), with myelin pallor and loss of calbindinD_28k_-labeled olivary efferents in the hilum (**b**, **f**). Residual neurons in the principal olive are often atrophic (small black arrows, **d**). Olivary neurons and GAD+ nucleo-olivary staining are preserved in the medial accessory olive (black arrow in **a**, **b**, **e**, **f**, **g**, **h**). GABA-ergic nucleo-olivary afferents show some mild patchy loss predominantly in dorsal and lateral regions of the principal olive (**h**). The dorsal arm in the principal olive shows a region with more numerous calbindinD_28k_-labeled neurons and preserved GAD staining (red arrow, **f**, **h**, **j**). Scale bars: 250 μm, A, B, E, F, G, H; 200 μm, top panel in C and D, I, J; 50 μm, bottom panel in C and D

Olivary degeneration was marked (Fig. 4a-d) and bilateral. The dorsal and ventral arms of the principal olive show marked and uneven neuronal loss, highlighted by immunostain to calbindin_D28k_ (Fig. 4e–j). The residual olivary neurons were atrophic (Fig. 4d, small black arrows) and surrounded by gliotic parenchyma. Quantification of ION neuronal linear density revealed marked neuronal loss, being only ~25% of the values previously determined in ET cases and controls (Table 1), and consistent with myelin pallor in LH&E stain and loss of calbindin_D28k_ labeled olivo-cerebellar fibers in the hilum of the principal olive (Fig. 4b and f). In contrast to the principal olive, neurons in the medial accessory olive (black arrows in Fig. 4a–f) and a medial segment in the ventral arm of the principal olive were preserved (Fig. 4f). The reciprocal nucleo-olivary innervation from GAD+ GABA-ergic neurons in cerebellar nuclei was relatively well preserved in the patient’s olivary nuclei, although there was some irregular patchy synaptic loss more prominent in parts of the dorsal arm and lateral bend of the principal olive at this medullary level (Fig. 4g and h), indicating some loss of these neurons in regions of the dentate nucleus.

**Fig. 5:**
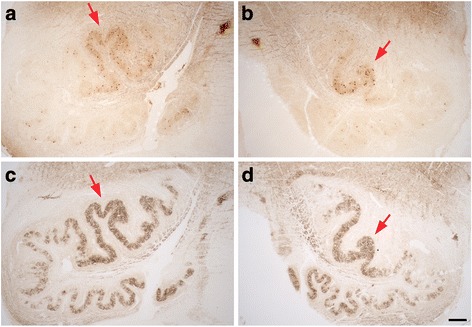
Degenerative changes in the patient’s left (**a**, **c**) and right (**b**, **d**) ION at a more rostral level, with immunostains to calbindinD_28k_ (**a**, **b**) and GAD (**c**, **d**). A distinct region with more numerous calbindinD_28k_-labeled neurons is again seen in the dorsal arm of the principal olive bilaterally along with preserved GAD staining (red arrows). There is patchy loss of GAD staining, more prominent in lateral and ventral regions of the principal olive in this region of ION. Scale bar: 250 μm, A-D

In the dorsal arm of the principal olive in this patient, there was a region with somewhat greater preservation, seen in both calbindin_D28k_ and GAD immunostains (Fig. 4f–h, red arrows). In an adjacent more rostral level of the medulla, this region of relative neuronal preservation was even more striking, and present bilaterally (Fig. 5a-d, red arrows). Loss of GAD+ GABA-ergic nucleo-olivary afferents was more prominent in this section, particularly involving lateral and ventral regions of the principal olive (Fig. 5c and d).

## Discussion and conclusions

Our patient had ET that was (1) of longstanding duration (i.e., from age 10 to death at age 94), (2) of childhood-onset (beginning at age 10), (3) clinically-progressive until death (Fig. 1), and (4) familial (i.e., by the patient’s report, two first-degree relatives were also affected). The features of the action tremor were typical of ET, with kinetic tremor greater than postural tremor [40] and there were no accompanying clinical features of parkinsonism or dystonia.

This case is similar in some respects to that reported by Elkouzi and colleagues [24]. Their patient had longstanding ET and then in later life developed degeneration of the ION. Despite this, their patient’s tremor did not change. The two cases differ in the sense that the types of changes in the ION were not the same – the published cases had pseudo-hypertrophic olivary degeneration [24] and ours had olivary degeneration of indeterminate cause. Also, our case came to postmortem, whereas the olivary changes in the published case were noted on brain imaging alone [24]. As discussed by Elkouzi and colleagues [24], these cases argue against the ION hypothesis of ET. The tremor in their case stayed the same after the onset of olivary degeneration. In our case, the age of onset of olivary degeneration is not known, but regardless, the tremor was documented to worsen, even during the final years of life, during which time, olivary degeneration must have been present (Fig. 1).

In postmortem studies of ET cases and controls, changes in the ION have not been detected [16, 41]. Moreover, in a detailed postmortem study of 14 ET cases and controls, a series of metrics was used to quantify microscopic neuronal and glial changes in the ION and its input and output tracts [23]. ION linear neuronal density also was assessed [23]. Cases and controls did not differ from one another with respect to any of the assessed metrics, and linear neuronal density also was similar in both groups [23]. In another study, the authors used vesicular glutamate transporter type 2 immunohistochemistry to label climbing fiber-Purkinje cell synapses in 12 ET cases and 13 controls [42]. Compared to controls, ET cases had decreased climbing fiber-Purkinje cell synaptic density, more climbing fibers extending to the outer portion of the molecular layer, and more climbing fiber-Purkinje cell synapses on the thin Purkinje cell spiny branchlets [42]. The interpretation of these findings is unclear. One possibility is that the abnormal climbing fiber-Purkinje cell synaptic connections in ET could be secondary to Purkinje cell degeneration while another is that it is due to a primary process involving the ION itself [42].

The cerebellar cortex was characterized by a range of degenerative changes (Fig. 2, Table 1), as have been described in patients with ET – an increase in torpedoes [16, 43] and an abundance of numerous other Purkinje cell axonal changes (e.g., increased axonal branching and axonal recurrent collaterals) [14], an increase in Purkinje cell dendritic swellings [44] and heterotopic Purkinje cells [45, 46], and marked hypertrophy of basket cell axonal processes [17]. These changes were within the range of what has been reported in patients with ET (Table 1). Additionally, there was marked Purkinje cell loss that was more severe than typically reported in patients with ET, as well as morphologic changes in the dentate nucleus largely reflecting loss of Purkinje cell afferents, only reported to some extent in two prior ET cases [16], indicating that the degree of degeneration in the cerebellum in this patient was in some respects even greater than that seen in ET. In cerebello-olivary degenerations, degenerative changes in the olive are presumed to be secondary to retrograde degeneration due to loss of Purkinje cell targets, and appear to directly correlate with disease duration [47, 48], although direct contribution from olivary climbing fibers to Purkinje cell degeneration cannot be fully excluded.

Our case did not exhibit intention tremor during the finger-nose-finger maneuver and there was no dysarthria or dysmetria of hand movements. There was mild dysmetria on the knee-tibia test on both sides. Mild problems with limb coordination and even frank ataxia have been reported in ET [6, 49, 50].

The cerebellum is organized in modules defined by 1) distinct parasagittal Purkinje cell zones projecting to defined cerebellar or vestibular nucleus regions, 2) the climbing fiber input from a subdivision of the contralateral inferior olive with a collateral projection to the same cerebellar target nucleus region, and 3) reciprocal connections of this target nucleus with the contralateral inferior olive by GABA-ergic nucleo-olivary neurons [38, 51]. The morphologic findings in this patient demonstrate interesting regional patterns of neuronal loss in cerebellum and inferior olive that correlate with this modular organization. The preserved Purkinje cells in the inferior vermis (not shown) and ION neurons in the medial accessory olive and a medial segment of the ventral principal olive in this patient (Fig. 4f) correlate with the somatotopic relationship between these areas [38, 52]. Consistent neuronal loss in dorsomedial regions of ION (Figs. 4 and 5) correlates with marked gross atrophy and neuronal loss in superior vermis in this patient, with similar Purkinje cell and granule cell loss as demonstrated for motor cerebellum (data not shown). There was also gross atrophy in lateral cerebellum of the posterior lobe, correlating with neuronal loss in lateral and ventral regions of the principal olive. The region in the dorsal principal olive with both less neuronal loss and preserved GABA-ergic nucleo-olivary input (Figs. 4 and 5, red arrows) is somatotopically connected with anterior lobe and adjacent posterior quadrangulate lobule in humans, suggesting there is a cerebellar module with relatively more intact circuitry, and likely less severe Purkinje cell loss, involving a region of motor cerebellum. Last, intact GABA-ergic nucleo-olivary synaptic staining did not always clearly correlate with the degree of ION neuronal preservation. Thus, parts of the circuitry in cerebellar modules may variably persist and/or be lost, and integrity of ION neurons appears to more prominently depend on preservation of olivary climbing fiber connections with Purkinje cells; a proposed trophic role of GABA-ergic afferents for survival of ION neurons could potentially contribute [53, 54].

The patient also had incidental Lewy bodies. On alpha synuclein stain, one 100× microscopic field of the basal region of the amygdala included five Lewy bodies but otherwise Lewy body containing neurons were rare in the amygdala and not found in the substantia innominata, substantia nigra pars compacta, locus ceruleus, or elsewhere on alpha synuclein stain.

The current case study should be interpreted within the context of several limitations. First, as with any case study, this was an n of 1. Additional cases are needed to confirm and elaborate upon our data. Second, we do not know exactly when the changes in the ION began in our case. There was no obvious clinical correlate (e.g., myoclonus or ataxia); presumably, though, it was a later-life occurrence.

In summary, although the pathophysiology of ET is not completely understood, evidence such as this suggests that the ION does not play a critical role in the generation of tremor in ET patients.

## Abbreviations

CERAD: Consortium to Establish a Registry for AD
ET: Essential tremor
ION: Inferior olivary nucleus
LH&E: Luxol fast blue counterstained with hematoxylin-eosin
